# Challenges in understanding psychiatric disorders and developing therapeutics: a role for zebrafish

**DOI:** 10.1242/dmm.019620

**Published:** 2015-07-01

**Authors:** Jasmine M. McCammon, Hazel Sive

**Affiliations:** 1 Whitehead Institute for Biomedical Research, Nine Cambridge Center, Cambridge, MA 02142, USA; 2 Department of Biology, Massachusetts Institute of Technology, Cambridge, MA 02139, USA

**Keywords:** Zebrafish, Psychiatric disorders, Animal models, Co-morbidities, Chemical screens, Autism, Schizophrenia

## Abstract

The treatment of psychiatric disorders presents three major challenges to the research and clinical community: defining a genotype associated with a disorder, characterizing the molecular pathology of each disorder and developing new therapies. This Review addresses how cellular and animal systems can help to meet these challenges, with an emphasis on the role of the zebrafish. Genetic changes account for a large proportion of psychiatric disorders and, as gene variants that predispose to psychiatric disease are beginning to be identified in patients, these are tractable for study in cellular and animal systems. Defining cellular and molecular criteria associated with each disorder will help to uncover causal physiological changes in patients and will lead to more objective diagnostic criteria. These criteria should also define co-morbid pathologies within the nervous system or in other organ systems. The definition of genotypes and of any associated pathophysiology is integral to the development of new therapies. Cell culture-based approaches can address these challenges by identifying cellular pathology and by high-throughput screening of gene variants and potential therapeutics. Whole-animal systems can define the broadest function of disorder-associated gene variants and the organismal impact of candidate medications. Given its evolutionary conservation with humans and its experimental tractability, the zebrafish offers several advantages to psychiatric disorder research. These include assays ranging from molecular to behavioural, and capability for chemical screening. There is optimism that the multiple approaches discussed here will link together effectively to provide new diagnostics and treatments for psychiatric patients.

## Abstract (precis)

**Summary:** In this review, we discuss strengths and limitations of prevalent laboratory models that are used for understanding psychiatric disorders and developing therapeutics, with emphasis on the zebrafish.

## Introduction

Accounting for nearly a quarter of global disability, psychiatric disorders represent a pervasive societal challenge (Whiteford et al., 2013), making the need to precisely diagnose and treat these disorders urgent. Psychiatry faces special challenges compared with other branches of medicine. Whereas twin and family studies have clearly demonstrated that psychiatric disorders are heritable, recent data reveal that the genetics of psychiatry are very complex (Kendler, 2013). A first major challenge for psychiatry, then, is to link molecular and cellular alterations to changes at the genome level – the ‘genotype’. Hundreds of loci in the human genome have been implicated in major psychiatric disorders, such as autism spectrum disorder, bipolar disorder and schizophrenia (Koboldt et al., 2013; Schreiber et al., 2013). Moreover, many of these loci are shared between different psychiatric disorders (Cross-Disorder Group of the Psychiatric Genomics Consortium and Genetic Risk Outcome of Psychosis Consortium, 2013), suggesting commonalities in dysfunction. These generally polygenic disorders are thought to arise from an array of gene variants that each contribute incrementally to disease risk (Gratten et al., 2014). For example, a genome-wide association study for schizophrenia estimates that around 8300 variants account for 32% of the heritability of schizophrenia (Ripke et al., 2013). With this high number of possible risk genes, determining whether gene variants are causally linked to a particular disorder remains difficult. A portion of autism spectrum disorder patients can be accounted for by various genetic syndromes tightly associated with known mutations and cytological anomalies (Caglayan, 2010). However, even in these Mendelian disorders, there is incomplete penetrance of autism and variable phenotypic presentations; for example, although fragile X syndrome is considered a syndromic form of autism, only around 30% of fragile X syndrome patients (who carry mutations in the *FMR1* gene) are diagnosed with autism (Fatemi and Folsom, 2011). This incomplete penetrance indicates that even when one predominant gene is associated with a disorder, other regions of the genome and/or the environment can modify its severity. Uncovering the complex genetics underlying psychiatric disorders and linking genotype to phenotype therefore remains a huge challenge.

A second major challenge for psychiatry is to improve diagnostics by defining the molecular, cellular and biochemical changes associated with each disorder – its ‘molecular pathology’. The behavioural criteria currently used to diagnose these disorders are powerful, but also complex, qualitative and sometimes subjective, in which independent tests of the same patient might not yield the same diagnosis (Lord et al., 2012). Molecular pathology of a disorder might include changes in gene expression, as well as changes in cell biology – including neuronal, glial or synaptic cell biology – defining biomarkers that are putatively causal to the disorder. The more closely associated the changes are to the key affected cells, the more robust the definition of molecular pathology. More distantly associated biomarkers, such as hormone levels in blood or urine, are also useful because they are easier to measure.

One complication that is encountered when defining molecular pathology is the presence of co-morbid, or co-occurring, disorders and symptoms, which might contribute to the overall pathology of a disorder (Kanner, 2013; Muir-Cochrane, 2006). For example, nearly 50% of people with a psychiatric diagnosis meet the criteria for two or more distinct psychiatric disorders (Kessler et al., 2005b). The genetic underpinnings of psychiatric and non-neural co-morbid symptoms are presumed to be linked, although this relationship has not been thoroughly addressed. Whereas some co-morbid symptoms impact the central nervous system (CNS), they can also affect other organs, leading to intestinal or immune dysfunction, obesity, hypotonia and cardiovascular disease (Hamdani et al., 2013; Kohane et al., 2012). Interestingly, individuals with disorders as behaviourally diverse as autism spectrum disorders (ASDs) and schizophrenia appear to share certain non-CNS symptoms, although it is not known whether a common molecular basis underlies this overlap.

Defining the molecular pathologies that underpin these disorders will allow for more precise diagnostics to be developed, and might establish subgroups of a disorder consistently linked to a specific biomarker. Another crucial outcome of defining the molecular changes that associate with psychiatric disorders is that these will help to identify new therapeutic targets.

Many current psychiatric medications were serendipitously discovered over 50 years ago. The targets of these drugs are unknown or not fully understood, and these medications are still used, despite significant side effects, often because there is no better alternative (Hyman, 2014). Therefore, a third major challenge for psychiatry is to identify new therapeutics. Although psychiatric medications account for the largest revenue in the pharmaceutical industry (Hyman, 2012), the funding and enthusiasm for psychiatric drug development is dwindling. A profit incentive is not enough to overcome the enormous cost of clinical trials and the high rates of failure. Although there is hope that patients, and therefore therapies, would fall into a limited number of categories, personalized approaches might be necessary because the genetics of psychiatric disorders is so complex. This possibility extends the challenge of therapeutic development in that genotype and phenotype will have to be linked on a per-patient basis.

**Fig. 1.**
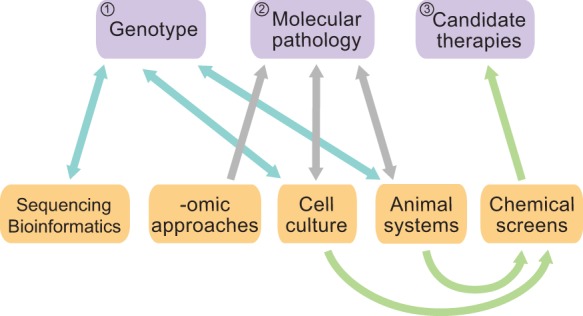
**Research strategy and integrative approach for overcoming the three major challenges of psychiatry.** For psychiatric disorders, there is no direct path from diagnosis to treatment. Three major challenges (purple) must be overcome by the use of different assays (orange). (1) First is the challenge of correct genotype identification, which can be determined by sequencing and bioinformatics methods, and using cell culture and animal systems to study disease-associated gene variants (blue arrows). Patient-associated genotypes can also be used to generate cell culture and animal tools. (2) The use of -omic approaches (such as metabolomics, proteomics and transcriptomics), cell culture and animal systems can provide clues about pathology (grey arrows). Determining the molecular pathology can improve diagnostics. (3) Cell culture and animal models can be used in chemical screens to identify candidate drugs (green arrows). Overall, this illustration demonstrates that in psychiatry the route from genotype identification to the understanding of molecular pathology to treatment is not direct but varied and complex, with cellular and animal models playing an important role.

In overcoming these three major challenges, it is important to determine which experimental tools would most effectively address them. There is no direct route from diagnosis to a new therapeutic without intervening experimental analysis (Fig. 1). Based on successful approaches used in a collaborative and iterative fashion in other disorders such as cancer (Rudrapatna et al., 2012; White et al., 2013), in which the molecular details are better understood, multiple approaches will be essential to improve the diagnostics and treatments for psychiatric disorders. Together with clinical input, useful approaches include sequencing and bioinformatics analysis, -omic approaches such as proteomics and metabolomics, cell culture assays and whole-animal assays. We focus the remainder of this Review on a few pivotal cellular and whole-animal approaches to address these three major challenges of psychiatric disorders (attributes summarized in Table 1), including neuronal and cell culture, induced pluripotent stem cell culture, cerebral organoids, *C. elegans*, *Drosophila* and mice*.* We conclude with an emphasis on the zebrafish, a laboratory organism with tools and attributes to study a wide range of psychiatrically relevant genetics and pathology, and a key vertebrate for chemical screens.

**Table 1.**
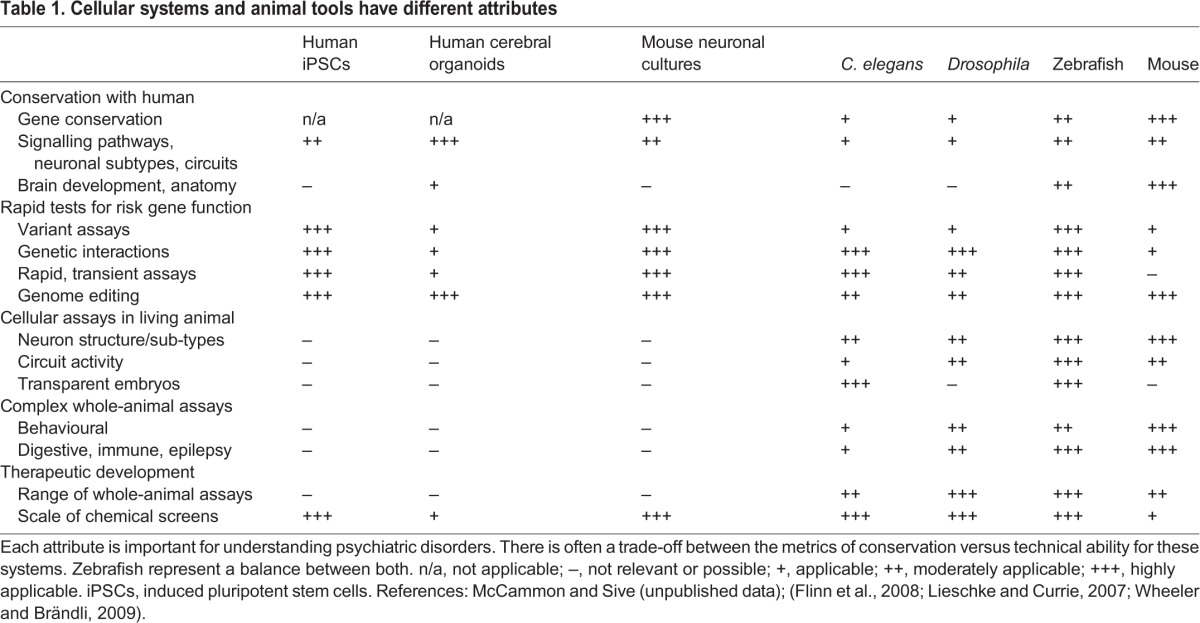
**Cellular systems and animal tools have different attributes**

## Cellular tools for psychiatric disorder research

Cell cultures, ranging from immortalized cell lines to patient-derived induced pluripotent stem cells (iPSCs), offer researchers and clinicians a high-throughput manner in which to study cellular pathology associated with psychiatric disorders. This approach is also invaluable for screening large chemical compound libraries for potential therapeutics that can reverse these cellular pathologies. In the case of iPSCs, patient-specific genetic variation is replicated for comprehensive study of genetic effects on cellular pathology. Furthermore, iPSCs can be differentiated to form three-dimensional organoids resembling rudimentary forms of organs, to facilitate the study of whole organ pathology *in vitro* (Box 1).

### Cell culture approaches

Both non-neuronal and neuronal cell culture are useful for elucidating the genetic basis of a disorder and for assessing the pharmacologic effects of potential therapies at the cellular level. For instance, the toxicity, receptor binding and dopamine transport-inhibition properties of Ritalin have been measured in human and hamster cells, as well as the expression levels of the dopamine transporter gene variants associated with attention deficit hyperactivity disorder (ADHD) (Grünblatt et al., 2013). At the level of neural circuits, the development of mammalian cortico-striatal cultures has facilitated analysis of synaptic activity. By mixing dissociated mouse neurons from cortex and striatum, synaptic connections that form can be studied and manipulated genetically or pharmacologically (Randall et al., 2011).

The development of iPSCs derived from patient biopsy, usually of the skin, has offered a unique system to study cellular pathology related to psychiatric disorders. When differentiated into neurons, iPSCs offer a way to study human cellular phenotypes, and can even form simple circuits in culture (Shi et al., 2012), in the context of the patient's genetic background. iPSCs have revealed clues about pathology and potential treatments: for example, iPSC-derived neurons from Rett syndrome patients show reduced synaptic puncta and decreased glutamatergic signalling, deficits rescued by insulin-like growth factor 1 (IGF1) application (Marchetto et al., 2010). IGF1 treatment has shown preliminary signs of ameliorating some breathing and behavioural abnormalities in patients (Khwaja et al., 2014). A screen of 1000 compounds using human iPSC-derived neural progenitor cells identified five compounds that could increase the proliferation or viability of these cells, hinting at their potential therapeutic use for modulating neuronal stem cells (McLaren et al., 2013), an approach that could prove useful for psychiatric disorders associated with dysregulated neurogenesis, including autism, schizophrenia, bipolar disorder and depression (Dodd et al., 2013; Wei et al., 2014). Cell culture and iPSC-derived neurons can also be used for high-throughput analyses; however, their application is limited to cellular phenotypes. In addition, iPSC-derived neurons do not progress developmentally beyond foetal-type stages (Brennand et al., 2014).

### Cell culture approaches: organoids

One surprising finding over the past few years is that stem cells can organize themselves *in vitro* into mini organs (Li et al., 2014). Recently, specialized cultures of iPSCs have generated cerebral organoids, mini-embryonic brain-like structures (Lancaster and Knoblich, 2014), with implications for addressing psychiatric disorders (Box 1). Cerebral organoids derived from a microcephalic patient with a mutation in cyclin-dependent kinase 5 regulatory subunit associated protein 2 (*CDK5RAP2*) were smaller than those derived from control iPSCs (Lancaster et al., 2013). Mouse models carrying mutations in the same gene do not show microcephaly, demonstrating the importance of using human tissue in this analysis. Studying organoids allows for the discovery and characterization of phenotypes that might not develop in two-dimensional cell cultures. Organs presenting co-morbid phenotypes or susceptibility to drug toxicity, such as the intestine and liver, can also be cultured as organoids (Spence et al., 2011; Takebe et al., 2013). At present, cerebral organoids are highly variable in phenotype and do not recapitulate the precise organization of the brain; however, the promise of this system is considerable. In the longer term, this approach might reveal several aspects of a disorder phenotype in a patient-specific genetic context and support the exploration of drug treatments (Ranga et al., 2014). Another significant limitation of the organoid culture is that the whole-animal phenotype is not recapitulated. We discuss this and other advantages of animal-based systems below.

**Box 1. Cell culture approaches**

Cell-based approaches:

- Challenges addressed: Genetic interactions and variant analysis, cell-autonomous phenotypes for biomarkers, therapeutic development for cellular phenotypes.
- Pros: Patient-specific genetic information, large scale, short time frame.
- Cons: No behavioural assays, no co-morbidity analysis, no system-wide or whole-organism screening.

Organoid approaches:

- Challenges addressed: variant analysis, organ-like phenotypes for biomarkers, therapeutic development.
- Pros: Patient-specific genetic information, three-dimensional (3D) structure.
- Cons: Immature or partial organs only; no ability for co-morbidity analysis, no systems/whole-organism toxicity screen.

## Whole-animal tools for psychiatric disorder research

Non-human animal systems are crucial for understanding phenotypes associated with a specific psychiatric disorder, allowing phenotypes to be investigated in the whole animal throughout its life. As human behaviours are often not faithfully modelled in animal systems (Nestler and Hyman, 2010; O'Leary and Cryan, 2013), whole-animal analyses might be most useful when they focus on specific molecular and cellular pathways that underlie a pathology. The concept of an animal ‘tool’, a biological system that can provide insight into a disorder without obviously recapitulating all associated phenotypes, is useful for pre-clinical studies (Sive, 2011). Numerous whole-animal systems have been used for analysing aspects of psychiatric disorders, including invertebrates, such as *C. elegans* and *Drosophila*, and vertebrates, such as zebrafish and mouse. To maximize the utility of different animals, one must consider the attributes and limitations of each (Box 2).

**Box 2. Whole-animal approaches**

Invertebrates:

- Challenges addressed: Genetic interactions and variant analysis, whole-animal phenotypes for biomarkers and co-morbidity analysis, large-scale therapeutic development.
- Pros: Whole-system/organism toxicity screen, large scale, short time-frame.
- Cons: Less evolutionary conservation, relevance to humans might be unclear.

Mice:

- Challenges addressed: single (or few) gene connections to phenotype, whole-animal phenotypes for biomarkers and co-morbidity analysis, very small-scale therapeutic development.
- Pros: Whole-animal screens, evolutionary conservation with human genome.
- Cons: Expensive, only small-scale assays feasible, long time-frame.

Zebrafish:

- Challenges addressed: genetic interactions and variant analysis, whole-animal phenotypes for biomarkers and co-morbidity analysis, mid- to large-scale therapeutic development.
- Pros: Conservation, whole-organism screens, multi-organ analyses, medium-scale screens and short time-frame.
- Cons: Less conserved genetically and morphologically than mammals, longer time-frame than invertebrates.

### Whole-animal approaches: invertebrates

Invertebrate models are cheap to maintain and have short life cycles, making them useful for rapid, large-scale analysis. For example, the nematode *C. elegans* is amenable to RNA interference (RNAi) screens, and has been used to identify targets of clozapine, one of the most effective drugs for treatment-resistant schizophrenia (McEvoy et al., 2006). Of 6656 genes analysed in triplicate, 40 candidate suppressors of clozapine-induced phenotypes were identified, including one targeting the ACR-7 subunit of a nicotinic acetylcholine receptor (nAChR), homologous to several human α-like nAChRs (Saur et al., 2013).

Invertebrate genes exhibit some evolutionary conservation with human genes. Homologues of ∼50% of human disease genes can be identified in *C. elegans* (Culetto and Sattelle, 2000). The fruit fly *Drosophila melanogaster* has identifiable homologues for 75% of human genes implicated in disease (Reiter et al., 2001). The *FMR1* gene that is mutated in fragile X, the leading cause of inherited intellectual disability, has a homologue in fruit flies*. Drosophila fmr1* mutants challenged the long-held dogma that neurodevelopmental disorders could not be treated because the aberrant circuitry that leads to behavioural dysfunction is set during development (Leblond, 1964). However, McBride and colleagues used metabotropic glutamate receptor (mGluR) antagonists to ameliorate behavioural deficits in a *Drosophila fmr1* mutant; these treatments worked well, whether administrated to embryos or adults (McBride et al., 2005). Although these results were replicated in *Fmr1* mutant mice (Dolen et al., 2007; Yan et al., 2005), clinical trials for mGluR antagonists failed to demonstrate sufficient efficacy for some behavioural parameters in human fragile X patients (Jacquemont et al., 2011). Whether this failure was due to a lack of translation of the data from invertebrates to humans or some design flaw in the clinical trials remains unknown. Despite caveats, invertebrates remain the highest-throughput organisms for which whole-animal phenotypes can be assessed.

### Whole-animal approaches: mice

Although low throughput in terms of experimental numbers and outcomes, there are several advantages to using mice as a model organism. These include genome conservation, in which homologues of 99% of human genes can be found in mouse (Mouse Genome Sequencing Consortium et al., 2002), and the availability of advanced genetic manipulation tools and complex behavioural assays, albeit for non-human behaviours. Mouse models have been developed for candidate schizophrenia risk loci, such as disrupted in schizophrenia 1 (*DISC1*) and 22q11.2 deletion syndrome, and for candidate autism risk loci, such as methyl CpG-binding protein 2 (*MECP2*) and 15q11-13 deletion/duplication syndromes (Amir et al., 1999; Bundey et al., 1994; Sandanam et al., 1997). These mice exhibit behavioural deficits, as well as neuroanatomical and neurophysiological anomalies (Robertson and Feng, 2011). By combining cellular and whole-animal approaches, pathological mechanisms can be revealed in human-mouse chimeras. In this strategy, human iPSCs are introduced into the mouse blastula, or human tissue is transplanted into older immune-deficient mice to generate human-mouse chimeras (Eckardt et al., 2011). Thus, the molecular identity of part of the tissue or organ of interest is human, but functions *in vivo* in the context of a whole animal. For example, grafting human glial progenitor cells onto neonatal mice resulted in enhanced long-term potentiation at the chimeric synapses, and enhanced learning and memory in the mice (Han et al., 2013). On a very small scale, using a few top candidates, mice can be used to test therapeutics. The *Mecp2* mouse mutant has been used to identify a potential treatment for Rett syndrome, involving the reversal of neurological deficits with IGF1 (Tropea et al., 2009). IGF1 is currently being tested in phase 2 clinical trials for Rett syndrome treatment (clinicaltrials.gov identifier: NCT01777542).

Despite these considerable attributes, mice have multiple limitations for use in the analysis of psychiatric disorders. Not only are they more expensive to maintain than non-mammalian laboratory animals, but small litter sizes render it difficult to obtain sufficient animals for large-scale analysis. Due to this lower number of obtained animals and the current emphasis on understanding brain anomalies in psychiatric disorders, mice are generally not utilized to study multiple organ systems and co-morbidities. The expense and small numbers also affect the manner in which drugs are tested in mice: generally pre-screening is conducted *in vitro* or *in silico*, or hypothesis-driven candidates are chosen without any screening (Fortney et al., 2012; Moy et al., 2014; Wang et al., 2014).

## Meeting the challenges with zebrafish

We now focus on the zebrafish, an animal model that balances experimental tractability and conservation with humans. In addition to being relatively cheap to raise and maintain, they also produce large quantities of embryos. The considerable attributes of the zebrafish for addressing psychiatric disorders include a high degree of molecular, cellular, morphological and developmental conservation; rapid transient genetic assays; genome-editing ability; live imaging; characterized behaviours; co-morbidity study; and amenability to chemical screens to define potential therapies (Box 3).

**Box 3. Advantages and limitations of using zebrafish for psychiatric disorder research**

Advantages:

- Conservation of human disease genes and brain development/anatomy.
- Rapid transient manipulations of gene expression and the rapid development of zebrafish yields phenotypic information in days.
- Live, non-invasive time-lapse imaging provides the easiest and best approach for studying dynamic cellular phenotypes *in vivo*.
- Accessibility of whole brain with the use of optogenetic tools allows neural circuits to be identified and their function investigated.
- Psychiatrically relevant behaviours can be assayed in zebrafish, such as sociability, anxiety, optokinetic response and conditioned behaviour.
- The development and function of conserved organ and organ systems can be assayed to identify co-morbidities, such as epilepsy and digestive and immune dysfunction.
- Conservation of signalling pathways, neuronal subtypes and neural circuits, the likely targets of drug therapies.
- High fecundity, small size, ability to be arrayed on 96-well plates and availability of automated analysis tools make zebrafish a pre-eminent vertebrate system for high throughput chemical screens.

 Limitations:

- Caveats to transient loss-of-function analysis.
- Partial genome duplication creates potential functional redundancy for some genes.
- Moderate length generation time.
- Trade-off in chemical screens between space for larvae and behaviours that can be assessed.

### Defining genotype

Zebrafish and mammalian genomes are well conserved, with more than 80% of human disease genes represented in the zebrafish genome (Howe et al., 2013). Short-term molecular genetic manipulation can be achieved by simple microinjection, using morpholino-modified antisense oligonucleotides (MOs) (Nasevicius and Ekker, 2000) or small interfering RNA (siRNA) (De Rienzo et al., 2012) for loss-of-function (LOF) studies, and mRNA overexpression for gain-of-function studies. Although MO technology is helpful, MOs might have off-target effects, such as non-phenotypes that include activation of p53, which can be suppressed with p53 MO (Robu et al., 2007). Stringent criteria can ensure MO phenotypes are specific: these include preventing an MO phenotype with co-injected target RNA (engineered to lack the MO binding site), using two different MOs per gene, and demonstrating changes in endogenous RNA splicing with splice site targeting MOs (Eisen and Smith, 2008). Concerns have been raised regarding differences between mutant and morphant phenotypes (Kok et al., 2015); however, compensatory changes in the transcriptome might be present in mutants but not in morphants, perhaps contributing to the differences (Stainier et al., 2015). At present, MO technology therefore remains useful. Human and zebrafish gene function is often interchangeable, and introducing a human gene (by mRNA injection or as a transgenic line) to replace the zebrafish version to ‘humanise’ the fish is valuable for analysing the function of human gene variants (Singh et al., 2011).

Single-gene studies can be conducted in other systems as well, so considering the advantages of using zebrafish for genotype studies would be prudent. One such advantage is rapid single-gene LOF analyses of copy number variant (CNVs) genes. CNVs are segments of the genome that have been duplicated or deleted, and they have been significantly associated with psychiatric disorders (Grayton et al., 2012). However, as deleterious CNVs often encompass multiple genes, the challenge is to determine which of these genes links the CNV to the disorder. Our group and others have used zebrafish to investigate the 16p11.2 CNV genes, in which deletions and duplications account for ∼0.8% of ASD cases, and duplications account for ∼0.35% of schizophrenia cases (Malhotra and Sebat, 2012), making this the most significant CNV for psychiatric disorders. Different assays were chosen as a readout of gene function, and different genes were reported as crucial: aldolase A (*aldoa*) and kinesin family member 22 (*kif22*) for early brain morphology, and potassium channel tetramerization domain containing 13 (*kctd13*) for larval head size, implying that multiple genes from this CNV affect several processes (Blaker-Lee et al., 2012; Golzio et al., 2012). Another advantage of zebrafish is their use for analysing gene-gene interactions. Rapid, MO-based, LOF assays have highlighted many examples of zebrafish gene interactions that inform our understanding of human disorders. For instance, a pairwise analysis of multiple genes associated with Bardet–Biedl syndrome – a pleiotropic intellectual disability disorder characterized by many symptoms, including cilia dysfunction and polydactyly – identified eight novel genetic interactions affecting a ciliated patterning organ and fin bud patterning/fin skeletal elements (Tayeh et al., 2008). Given the polygenic nature of many mental health disorders, zebrafish have great utility for the rapid study of genetic interactions.

Such gene manipulation approaches also allow for the investigation of human gene variants, identified by sequencing patient genomes. Determining whether these variants are causal to a disorder is challenging, because the signal-to-noise ratio can be quite low for common variants with low penetrance. Zebrafish studies can complement bioinformatics predictions about function (Samocha et al., 2014), helping to sort out which variants have wild-type and which have abnormal activity. For example, Gauthier and colleagues showed that a rare variant of SH3 and multiple ankyrin repeat domains 3 (*SHANK3*) associated with schizophrenia could not rescue head size and swimming deficits in a zebrafish *shank3* LOF embryo, whereas a second variant and wild-type RNA could (Gauthier et al., 2010), suggesting that the first variant was pathological. For the schizophrenia risk locus *DISC1*, variants were identified from patient pools and tested in *Disc1* LOF mouse embryos to determine which could and which could not rescue neuronal progenitor proliferation. When they were injected in *disc1* LOF zebrafish embryos, the variants that showed maintenance or loss of activity in mice exhibited similar patterns in rescuing or not, respectively, brain ventricle and axon tract defects in zebrafish embryos (Singh et al., 2011). These results emphasize the conservation of variant function between the two species, and indicate that a much higher number of variants can be analysed in zebrafish than is feasible in the mouse.

Targeted gene knockout and transgenic methods have also been developed for zebrafish; thus, single or multi-genic gain- or loss-of-function mutants can be efficiently produced to define gene interaction networks and novel regulatory connections. Genes can be mutated by chemical or insertional mutagenesis (Amsterdam and Hopkins, 2006). More recently, naturally occurring pathogen defence mechanisms in plants and bacteria have been co-opted to develop targeted genome-editing tools for zebrafish, including transcription activator-like effector nucleases (TALENs) and the clustered regularly interspaced short palindromic repeats (CRISPR)/CRISPR associated protein 9 (Cas9) system, to generate mutations in genes of interest (Hisano et al., 2013). CRISPRs targeting five different genes have been injected into single zebrafish embryos and induced mutations at all five loci (Jao et al., 2013).

Finally, zebrafish are amenable to genetic tricks such as generation of haploids, which are viable for about two days, or gynogenetic diploids – the generation of diploid animals from only the maternal genome – which are completely viable. This feature makes potential screens faster and more efficient as recessive mutations are revealed in these lines without the need for further crossing. For example, a zebrafish genetic suppressor screen of 800 haploid genomes in a conditional haematopoiesis mutant identified two suppressors (Bai et al., 2010). In summary, zebrafish genetics are well suited for bulk transient analyses, particularly for variant analysis and genetic interactions.

### Defining molecular phenotypes associated with psychiatric disorders

Relating genotype to psychiatrically relevant phenotypes in laboratory animals has the caveat that human-specific behaviours define mental health disorders. However, endophenotypes, that is, biochemical, cell biological or molecular markers, are associated with psychiatric disorders (Gottesman and Gould, 2003) and might define underlying mechanism. Zebrafish are well suited to rapidly assess a wide range of potential endophenotypes (Norton, 2013).

With the variety of tools available for transient genetic manipulation in rapidly developing embryos, researchers studying zebrafish can complete phenotyping assays in just a few days. By 24 hours of age, a zebrafish embryo has formed all its major organs, allowing many phenotypic assays to be employed. Patterns of neurogenesis in the zebrafish CNS are generally similar to those found in mammals (Panula et al., 2010), and the overall architecture of the zebrafish brain closely resembles that of the mammalian brain, although teleosts do not have a neocortex. The zebrafish telencephalon develops by eversion rather than invagination, resulting in somewhat different positioning of telencephalic structures; however, the structures are conserved (Wullimann, 2009). Whereas the prefrontal cortex is known to play a considerable role in psychiatric disorders, the cerebellum has also been strongly implicated in their aetiology (Reeber et al., 2013). It is therefore relevant that the anatomy and development of the human and zebrafish cerebellum are highly conserved (Hashimoto and Hibi, 2012).

Psychiatric disorders are increasingly referred to as ‘synaptopathies’ because of the presence of drug targets, risk-gene candidates and aberrant signalling at the synapse (Grant, 2012). Due to the plasticity of synapse generation and function, more can be learned from observing synapses in intact circuits *in vivo* than from fixed, post-mortem samples. The transparency of the zebrafish embryo supports live imaging at single-cell resolution to develop cellular assays of neuropathology. For example, using fluorescent labels, time-lapse microscopy has enabled researchers to examine dendritic arborisation *in vivo*. This included evaluating the changing dynamics for pre-synaptic activity, competition from neighbours affecting outgrowth and the turnover of synapses in zebrafish (Ben Fredj et al., 2010; Meyer and Smith, 2006). These assays offer unprecedented neural function analysis in the living animal.

Integral to understanding the pathology that underlies psychiatric disorders is the characterization of neuronal circuits. The zebrafish is well suited for this, as transgenic lines are straightforward to create, and hundreds of GAL4 enhancer trap lines have been generated with varying neural expression patterns. In this approach, the gene encoding the yeast transcription activator protein GAL4 is expressed in only a subset of neuronal cells. These GAL4 lines are then crossed with zebrafish transgenic lines harbouring fluorescent reporter genes downstream of an upstream activating sequence (UAS) to which GAL4 specifically binds; this will drive the expression of the fluorescent reporter and thus make this subset of neuronal cells visible (Asakawa and Kawakami, 2008; Scott et al., 2007). Because of its external fertilization, transparency and small brain size, the larval zebrafish brain is nearly entirely accessible to two-photon stimulation and to activity readouts without any invasive surgery. Optogenetic tools for manipulating neuronal activity and fluorescent indicators of calcium activity (such as GCamP) have been used to dissect circuit components for behaviours in zebrafish (Fajardo et al., 2013; Kimura et al., 2013; Wyart et al., 2009). These approaches have revealed that the saccade-generating neurons of the optokinetic response (which is when the eyes track a moving object until it exits in the field of view and then reset to where the movement entered the field of view), a behaviour that is defective in several psychiatric disorders, are located in a small area of the zebrafish hindbrain in rhombomere 5 (Schoonheim et al., 2010).

Linking molecular pathology to behaviour might be the most challenging step in understanding psychiatric disorders. Whereas zebrafish do not generally phenocopy human behaviour, they do exhibit a repertoire of stereotypic behaviours with potential psychiatric relevance that could be useful indicators of gene function (Brennan, 2011; Stewart et al., 2013). These behaviours include sociability, anxiety, the optokinetic response and conditioned behaviour. Social aberrations are a defining symptom of ASDs but can also be noted in schizophrenia and mood disorders. Zebrafish are a good system to examine sociability because they spend 90% of their time in a shoal or in loose groupings (Buske and Gerlai, 2012). When zebrafish are treated with MK-801, a compound used to induce autistic and schizophrenic behaviours in rodents (Neill et al., 2010), they exhibit disrupted shoaling behaviours (Maaswinkel et al., 2013). Anxiety is a co-morbidity for psychiatric disorders; nearly 30% of Americans will suffer from some anxiety disorder in their lifetimes (Kessler et al., 2005a). Defects in glucocorticoid signalling are associated with depression and psychosis (Pariante, 2009). Similarly, a zebrafish glucocorticoid receptor mutant shows increased anxiety in the novel tank diving assay: these fish freeze and show reduced exploratory behaviour, which can be rescued with the addition of antidepressants to their holding water (Ziv et al., 2013). Furthermore, optokinetic response deficits are associated with several psychiatric disorders (Bittencourt et al., 2013; Trillenberg et al., 2004). SNPs near the dopa decarboxylase gene (*DDC*) have been significantly associated with autism (Toma et al., 2013), and *ddc* knockdown in zebrafish results in a defective optokinetic response (Shih et al., 2013). Finally, assaying conditioned behaviour in zebrafish can probe aberrations in cognitive function, learning deficits and memory, all of which have been tied to psychiatric disorders. After being conditioned with shock stimuli, zebrafish mutant for the fragile-X-associated gene *fmr1* show decreased latency to enter the shock zone of their tanks compared with wild-type fish, implying that they have a deficit in inhibitory avoidance learning (Ng et al., 2013).

Also integral to defining pathology is identifying the co-morbidities associated with particular psychiatric disorders. Zebrafish exhibit similar physiology to humans in digestive and immune function (Renshaw and Trede, 2012; Shepherd and Eisen, 2011), both of which can be compromised in psychiatric patients (Hamdani et al., 2013; McElhanon et al., 2014). Intestinal function can be monitored in zebrafish by utilizing microgavage and fluorescent beads or by liposome incubation (Carten et al., 2011; Cocchiaro and Rawls, 2013). Seizure behaviours, which are also often associated with psychiatric disorders, have been studied extensively in zebrafish as well (Baraban et al., 2005; Stewart et al., 2012). The transparency of these fish, the availability of transgenic reporter lines and behavioural analyses, and live assays of conserved neural circuit and organ function afford unique opportunities to study such phenotypes in zebrafish.

## Defining new therapies for psychiatric disorders using zebrafish

All known mammalian signalling pathways are conserved in zebrafish, and almost all are active in the zebrafish embryo as the brain and other organs begin to develop (Stuhlmiller and García-Castro, 2012; Wullimann, 2009). As many such pathways are likely to be implicated in psychiatric disorders and can be targeted by potential therapeutics, the zebrafish embryo and larva can act as a whole-animal test tube for chemical screens. For small molecule screens, the small size of zebrafish embryos and larvae, and the large numbers that can be obtained, make the zebrafish the most powerful whole-vertebrate tool. The system is also useful for monitoring the toxicity and side effects of drugs at the organismal level.

Chemical screens relevant to psychiatric disorders have been conducted in zebrafish. As aberrations in Wnt signalling and glucocorticoid signalling have been implicated in mental health disorders (Freyberg et al., 2010; Pariante, 2009; Voleti and Duman, 2012), zebrafish chemical screens have been conducted for modulators of β-catenin (Hao et al., 2013) and glucocorticoid signalling (Weger et al., 2012). In addition, screens for compounds that affect sleep have been carried out (Rihel et al., 2010), as circadian/sleep disturbances are often associated with psychiatric disorders (Dueck et al., 2012). Finally, by combining known and unknown psychotropic chemical libraries and using a behavioural readout, researchers used zebrafish to uncover novel classes of neuroactive compounds and predict their targets (Kokel et al., 2010).

Several zebrafish chemical screens have contributed to developing drugs that are currently in clinical trials. A compound that improves engraftment in umbilical cord hematopoietic stem cell transplants is currently undergoing clinical trial (Cutler et al., 2013; Goessling et al., 2011), as are a drug to kill melanoma cells (White et al., 2011) and a compound to treat Duchenne muscular dystrophy (Kawahara et al., 2011; Martin et al., 2012). Together, these examples indicate that chemical screens in zebrafish can produce effective and translatable results in the development of treatments for psychiatric disorders.

## Concluding perspectives

**Fig. 2.**
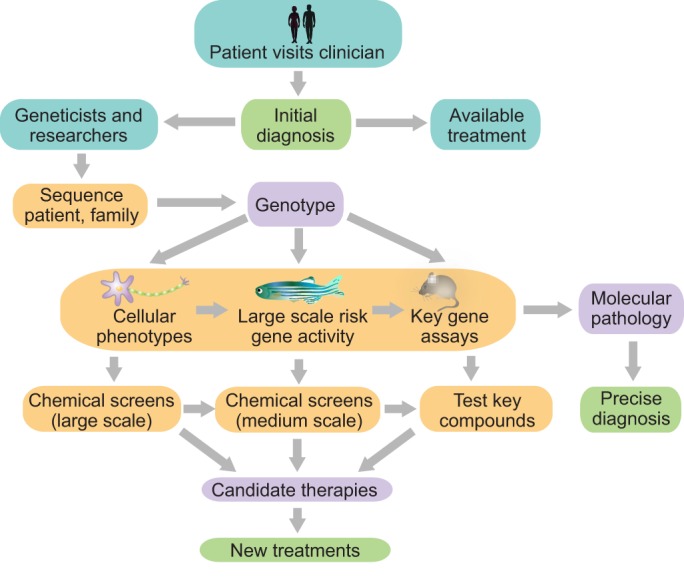
**Pragmatics of interfacing the patient with current research.** A patient visiting the clinic receives an initial diagnosis. Based on this diagnosis, the clinician can try available treatments or connect with geneticists and researchers. Due to the high heritability of some psychiatric disorders, exome sequencing of the patient and their family could identify variants associated with the disorder. Patient cells can be used to derive induced pluripotent stem cells (iPSCs) for studying cellular phenotypes. Mouse models are best used to study single, highly penetrant variants. Multiple variants or those of unknown function are productively screened in zebrafish for activity and interactions. A smaller number can then be analysed in mice. Cellular and animal assays reveal molecular pathology, which can lead to a more precise diagnosis. Zebrafish are also used to screen chemical libraries, or prioritized candidates from larger cellular screens, to identify modifiers of variant function in whole-animal assays. Key compounds can be analysed in mice. Top candidate therapeutics from these screens are tested in clinical trials to lead to the development of new treatments. Together, these approaches can identify patient genotypes, molecular pathology, more precise diagnostics and therapeutics. The main challenges of psychiatry are shown in purple, whereas the different assays that can help to overcome them are shown in orange. The desired outcomes of diagnostics and new treatments are shown in green.

How does this rich landscape of methodological possibilities integrate with psychiatric disorder diagnosis for a patient? We explore this in Fig. 2. After receiving a diagnosis from the physician, a patient will often want to try the available treatments. But patient and physician can also connect with a human geneticist to have the patient and unaffected family member DNA sequenced to identify putative, contributing risk genes. Depending on the candidate risk genes identified, different tools will give different deliverables in different time frames. These tools complement one another in terms of readouts and scale of chemical screens. Putting these approaches together maximizes the chance of effectively identifying causal genotype, diagnostics and therapeutics.

There are things a zebrafish cannot do. A zebrafish cannot show human autism or schizophrenia behaviours. But neither can a mouse. Some genes are not conserved and the zebrafish brain has only rudimentary cerebral hemispheres. All animal models have their limitations and must continually be evaluated as to whether they display phenotypes relevant to a psychiatric disorder. As we have explored, to define the molecular basis for each psychiatric disease, a substantial set of animal model and cell-based tools will be needed, and among these, the zebrafish is an effective and useful contributor.
